# Personalised 3D printed respirators for healthcare workers during the COVID-19 pandemic

**DOI:** 10.3389/fmedt.2022.963541

**Published:** 2022-08-01

**Authors:** Aidan D. Roche, Alistair C. McConnell, Karen Donaldson, Angus Lawson, Spring Tan, Kate Toft, Gillian Cairns, Alexandre Colle, Andrew A. Coleman, Ken Stewart, Paul Digard, John Norrie, Adam A. Stokes

**Affiliations:** ^1^Deanery of Clinical Sciences, Queens Medical Research Institute, The University of Edinburgh, Edinburgh, United Kingdom; ^2^School of Engineering, Institute for Integrated Micro and Nano Systems, The University of Edinburgh, Edinburgh, United Kingdom; ^3^Edinburgh Medical School, The University of Edinburgh, Edinburgh, United Kingdom; ^4^Roslin Institute, The University of Edinburgh, Edinburgh, United Kingdom; ^5^Department of Speech and Language Therapy, St John's Hospital, Livingston, United Kingdom; ^6^Department of Speech and Language Therapy, Royal Hospital for Sick Children, Edinburgh, United Kingdom; ^7^Bayes Centre, University of Edinburgh, Edinburgh, United Kingdom; ^8^Edinburgh Clinical Trials Unit, The University of Edinburgh, Edinburgh, United Kingdom

**Keywords:** COVID-19, PPE, 3D printing, facemask, fit-testing

## Abstract

Widespread issues in respirator availability and fit have been rendered acutely apparent by the COVID-19 pandemic. This study sought to determine whether personalized 3D printed respirators provide adequate filtration and function for healthcare workers through a Randomized Controlled Trial (RCT). Fifty healthcare workers recruited within NHS Lothian, Scotland, underwent 3D facial scanning or 3D photographic reconstruction to produce 3D printed personalized respirators. The primary outcome measure was quantitative fit-testing to FFP3 standard. Secondary measures included respirator comfort, wearing experience, and function instrument (R-COMFI) for tolerability, Modified Rhyme Test (MRT) for intelligibility, and viral decontamination on respirator material. Of the 50 participants, 44 passed the fit test with the customized respirator, not significantly different from the 38 with the control (*p* = 0.21). The customized respirator had significantly improved comfort over the control respirator in both simulated clinical conditions (*p* < 0.0001) and during longer wear (*p* < 0.0001). For speech intelligibility, both respirators performed equally. Standard NHS decontamination agents were able to eradicate 99.9% of viral infectivity from the 3D printed plastics tested. Personalized 3D printed respirators performed to the same level as control disposable FFP3 respirators, with clear communication and with increased comfort, wearing experience, and function. The materials used were easily decontaminated of viral infectivity and would be applicable for sustainable and reusable respirators.

## Introduction

The global COVID-19 pandemic has highlighted the extraordinary pressures on healthcare systems and on supply chains to produce sufficient respirators to protect healthcare workers. Additionally, as existing respirators which meet the WHO minimum filtering standard of Filtering Facepiece (FFP) 2 for aerosol generating procedures (1) do not fit all face types, it is common for staff to fail mandatory fit-tests for safe wear.

As a highly contagious viral disease, COVID-19 was originally thought to primarily spread through small droplets when an infected person coughed or exhaled, but recent evidence suggests it may be transmitted as aerosolised particles (2). While medical management of this disease continues to progress, infection prevention is the cornerstone of current policies in the form of vaccination and capable personal protective equipment (PPE). Healthcare workers are at particular risk of infection due to frequent and high dose exposure, and as such they need respirators that fit comfortably and are highly effective at filtering small particles. This need is demonstrated by evidence from China early in the pandemic where a lack of PPE led to 2,055 healthcare workers becoming infected, with 22 deaths (3), with the global burden becoming increasingly clear as the pandemic progressed (4).

With the rapid spread of the disease across the world, governments struggled to provide a constant supply of respirators to health workers in need, even when existing local manufacturing opportunities were sought (5). In this extreme situation, scientists, engineers and even hobbyists used 3D printing, amongst other open source techniques, to create respirators to help with the shortage but none of these have been validated for clinical use (6) respirator.

Individuals have unique facial anatomy with variations due to age, gender and ethnicity (7). Respirators provided in different sizes alone do not take into consideration this variation in anatomy, (8) and this lack of customization may result in high respirator fit-test failure rates, where the efficacy of airborne particles passing the filter and seal in a respirator is evaluated. Notably, there has been a higher prevalence of fit-test failures amongst clinicians who are female or in an ethnic minority within the UK (9, 10). The lack of safely fitting respirators has led to situations where qualified staff have been unable to work in clinical areas dedicated to managing COVID-19 patients.

3D facial scanning and model generation has the capacity to account for variations in facial anatomy (11). Using individual 3D face templates, 3D-printed respirators can be produced to conform accurately to specific facial features and reduce the risk of failing fit-tests. In addition, as 3D printers are now widely available, manufacturing of respirators can be done local to hospitals in need, helping to reduce the demand on overstretched supply chains.

Our study aimed to determine if personalized 3D printed respirators were as safe and effective as disposable FFP3 standard (confirming respirator filters 99% of all particles measuring up to 0.6 μm, with <2% inward leak) respirators with the potential to be used by healthcare workers. We demonstrate that problems of availability and fit can be overcome by using clinically available 3D scanning cameras and 3D modeling software, in combination with local 3D-printing technology, to produce FFP3 respirators which meet the highest standards of filtration, comfort, and speech intelligibility. Whilst this study provides evidence of this concept, it does not propose uptake in clinical environments without rigorous review by regulatory bodies.

## Methods

### Controlled trials

This study was conceived in response to the shortage of well-fitting respirators in our local region at the peak of the pandemic. Prior to submitting the study proposal, healthcare and occupational health staff at St John's Hospital were consulted on the alternatives to existing FFP3 supplies. The Occupational Health Department of St John's hospital assisted by providing local data on staff respirator fit-test results during the COVID-19 pandemic, and a small focus group of staff was assembled to discuss the “ideal” features of a respirator.

This single center, randomized, non-inferiority, controlled trial was conducted in one hospital in Scotland. Inclusion criteria were healthy healthcare workers in the NHS at the time of the study. The study took place between 1st October 2020 and 31st January 2021. Exclusion criteria were anyone considered vulnerable to COVID-19 as per national guidelines at the time of the study. Participants were required to be clean shaven as per local healthcare board policy for fit-testing. A minimum of 50 (21 male, 29 female) participants were recruited (Power = 0.8. alpha = 0.05), with a maximum of 62 to allow for participant withdrawals. Two participants withdrew from the study, and 10 participants did not complete the study either due to shift patterns or rotations to different hospitals preventing completion of at least one part of the study. Written or electronic consent was obtained from all participants before study enrolment. Each participant was assigned a randomized study number so they could not be identified during analysis by all investigators, except AR and AM. AR generated the random allocation sequence, KS enrolled the participants, and AR allocated the participants to each group. Participants were initially equally divided between two groups: one group undergoing 3D facial scanning and the other group having their 3D face model generated from three photographs as detailed further in the manuscript. Due to some participants not being able to complete the study, for the final analysis *n* = 27 were in the facial scanning group and 23 in the 3D face modeling group (Table 1 in Supplementary material).

Participants attended two sessions, at least 1 week apart with the control and the personalized 3D printed respirator, respectively. This gap in time was to allow any subjective experience the participants had with each respirator to fade. During each session, the participants took part in a respirator fit test, a communication intelligibility test, and a simulated clinical scenario while wearing a respirator to assess comfort, wearing experience and function. In addition, the participants were asked to wear both respirators at home for a period of 4 h to simulate and assess a longer wearing period.

### Experimental conditions

3030V respirators (Alpha Solway, Dumfriesshire, Scotland) were used as the control, based on Occupational Health records within St John's Hospital demonstrating these as the best available respirator, due to the highest amount of successful fit tests.

Each participant was randomly assigned to either have their face 3D scanned by the medical photography department within St John's Hospital using an Arctec Eva 3D scanner (Artec 3D, Luxembourg) or were asked to upload three self-taken photographs using their own mobile phones to an online machine learning 3D model generating application, Crisalix (Lausanne, Switzerland) (12). Each method produced a unique stereolithography (.STL) file for computer aided modeling and manufacture of the personalized 3D printed respirators. guidelines.

### Primary outcome measures

A PortaCount respirator quantitative fit tester 8038 (TSI Inc, Minnesota, USA) was chosen as it is widely used in clinical and industrial environments (13).

Fit-testing was in compliance with European standards BS EN 140:1999 (Respiratory protective devices. Half masks and quarter masks. Requirements, testing, marking) and BS EN 149:2001+A1:2009 (Respiratory protective devices. Filtering half masks to protect against particles. Requirements, testing, marking) respiratory protection standards, including performing a user-seal check prior to commencing the test.

Participants were shown how to don the respirator in accordance with the standard hospital procedure and then allowed to don the respirator themselves with the researcher on hand to provide any help or guidance if required.

Before each session began the PortaCount pre-checks were performed according to the manufacturer's guidelines. An aerosol generator was used to diffuse particles of sodium chloride into the ambient room atmosphere (provided by TSI Inc). Once the pre-checks were complete the PortaCount was placed into N95 mode and after 15 min of particle generation the aerosol concentration was checked to ensure it was at the required level for the fit testing and the manufactures calibration was performed.

Once the aerosol concentration was at the correct concentration a sampling probe was inserted into each respirator using a TSI Fit-Test Probe Kit. The Portacount would then perform a purge process to ensure any sodium chloride particulates were remove from within the respirator, probe and connection tube. After the purge was complete the fit test procedure could begin on each participant.

Each participant would perform a self seal test where once they had fitted their respirator (FFP3 or 3D printed) they would take a deep breath and blow out hard, checking using their hands underneath their chin and above the nose for any air leak, this would be repeated with the researcher repeating placing their hands under the chin and over the nose to detect and leaks.

Participants performed the following seven exercises: normal breathing, deep breathing, moving head side-to-side, moving head up and down, reading a passage aloud, bending at the waist, and repeated normal breathing as per the manufacturer's guidelines for 8038 model. Each exercise was performed in accordance to the time set by the TSI N95 mode, no alterations were made to the standard procedure. The set of exercises performed are in line with the St Johns Hospital fit testing procedure as this would provide a realistic comparison and it would also be familiar to all of the participants. We followed the standards set by the N95 mode on the PortaCount and at the St Johns Hospital where each of the fit tests exercises had to score a minimum of 100 out of 200 to pass, if any scored below the 100 the participants attempt was declared a fail and the PortaCount would stop. The final fit factor was taken as a summation of the seven exercises performed by each participant. The fit test was repeated for both the FFP3 and 3D-printed respirator.

The data was initially stored on the computer provided as part of the TSI PoraCount kit before being transferred to the University's secure data management system, all was anonymised in accordance with GDPR and the submitted ethical requirements.

### Secondary outcome measures

Clear verbal communication underpins effective team-based patient care. It is therefore key to evaluate the impact of respirator use on speech intelligibility. Speech clarity was assessed using a previously developed and evaluated psychoacoustic tool, the MRT, which uses lists of similar sounding words to assess speech intelligibility (14). Digital recordings of participants speech were anonymised and analyzed by two speech and language therapists, who were blinded to the respirator type. MRT scores were evaluated by the percentage of correctly heard words, with results confirmed between assessors to ensure agreement. These scores were then averaged, and compared between the respirator groups.

The R-COMFI is a 21-item psychometrically-sound measure of comfort and tolerability of filtering face-piece respirators (15). Participants completed R-COMFI questionnaires first following a short, high intensity simulated clinical scenario to assess respirator tolerability in a physically demanding environment [Clinical Simulation Suite, St John's Hospital at Howden]. Specifically, each participant worked in a small group of 3 to manage a simulated cardiac arrest, using a mannequin that could respond to questions *via* an operator, with all vital monitoring information provided on screens for 20 minin a clinical simulation suite designed to replicate a high dependency unit. A second questionnaire was completed following a 4 h lower intensity wear period at home to simulate a work shift. Participants were instructed to conduct the normal domestic activities, such as cooking, cleaning, gardening and taking breaks as required. No information was collected about specific tasks that the participants conducted at home.

To determine which 3D printing materials would be suitable for reuseable respirators and decontaminated using hospital protocols, virology testing was conducted. A total of ten different 3D printed materials and three different surface coatings were tested for effects on virus survival in the absence of disinfectants, all in comparison to a polystyrene material not known to be notably antiviral (16). Virus survival was assayed by sampling virus deposited on the various surfaces across a time course, from 0 h (≈around 10 min) to 24 h.

Viral stock of H1N1 strain influenza A virus A/Puerto Rico/8/1934 [PR8], as a surrogate for SARS-CoV-2, was grown in embryonated eggs as previously described (17). Plastic disks of potential 3D printed materials were sterilized by soaking in 70% ethanol for 5 min followed by air-drying overnight. For time course assays, 10 μl of viral stock was spotted on each disk, and at 0, 4, 8, 12, and 24 h after spotting, recovered by the addition of 990 μl of SFM supplemented with 1% BSA, snap frozen and stored at −80°C prior to titration by plaque assay.

To assess the ability of the materials to be disinfected by common cleaning agents, four materials and five cleaning agents were tested. The latter were: Chlor-Clean™ (the NHS standard disinfectant), 70% ethanol (as a readily available laboratory disinfectant) and hand sanitiser, shower gel and washing up detergent as disinfectants likely to be widely available in professional and domestic settings. Controls were polystyrene as a neutral surface and tissue culture medium (SFM) as a non-virucidal liquid.To test the disinfectants, 10 μl of viral stock was spotted on each disk as before and allowed to dry for ≈1.5 h. Following this, 50 μl of disinfectant: Chlor-Clean™ (Guest Medical, Aylesford, UK), 70% ethanol, hand sanitiser (Purell™; GOJO Industries, Milton Keynes, UK), shower-gel (diluted 1:10 with PBS), washing-up detergent or SFM [to serve as a negative control] was deposited on top of the dried viral spot. Five minutes after incubation, 940 μl SFM plus 1% BSA was used to recover the virus and the samples processed as before. Assays were set up in a minimum of triplicate.

Each participant's facial 3D model were gathered from either the Crisalix system (12) or Artec Eva 3D scanning The Artec Eva 3D system was performed by an experienced 3D scanning technician at St Johns Hospital. The Crisalix system used a H3D-Net hybrid scheme which combines model-based and model-free methods to generate an accurate 3D file with minimal input. Participants in the Crisalix group were required to use the Crisalix online system where they were asked to upload three photos of themselves (selfies). Each photo is checked by the Crisalix algorithm for light and angle and if a problem is encountered the user is requested to reupload their photos.

All of the *.stl* files from both the Crisalix system and the Artec Eva system were inspected in Meshmixer (Autodesk, California, USA). Two sections were created in Solid Edge (Siemens, Texas, USA) which would be used in combination with the individuals' *.stl* file to create the custom sections. One section was larger than the other section and used to create the lip of the respirator. Each section underwent a Boolean subtraction based on the participant's face and then combined to form the final custom section.

The manufacturing flow process can be seen in Figure 1 in Supplementary material fromt the point of .stl generation to the end customized respirator. An individual mold section for each face was first 3D printed, Replicator+ (MakerBot, NY, USA), uPrint SE (Stratasys, Rehovot, Israel), or PRO (Raise3D, California, USA). To correctly assign each customized respirator to each participant, their study number was etched onto their specific mask. The inverse mold was created by pouring Ecoflex™ 00–30 (Smooth-On) over the 3D printed section. Once the inverse mold was cured, Ecoflex™ 00-50 (Smooth-On) was poured in and a 3D printed standard part was placed into liquid silicone. Once curing was complete (≈3 h), the finished respirator was removed from the mold.

Two straps went from the upper section of the respirator linking to an adjustable 3D printed catch where they then split into two further straps which sat on the crown of the head. There was also an adjustable Velcro strap at the lower point of the respirator that went around the participant's neck.

For the filter material, we chose a disposable bacterial and viral filter material (Numed Healthcare, Sheffield, UK) commonly used in spirometry testing. The filter material had been tested for viral and bacterial filtration efficacy by the Nelson Laboratories and they had an efficacy of >99.99% for both bacteria and viruses (18).

Once all the sections were fabricated, the respirator was assembled. The filter housing was connected *via* a locking push fit to the front of the primary respirator section and then each of the straps were fed into their respective loops on the main respirator.

**Figure 1:**
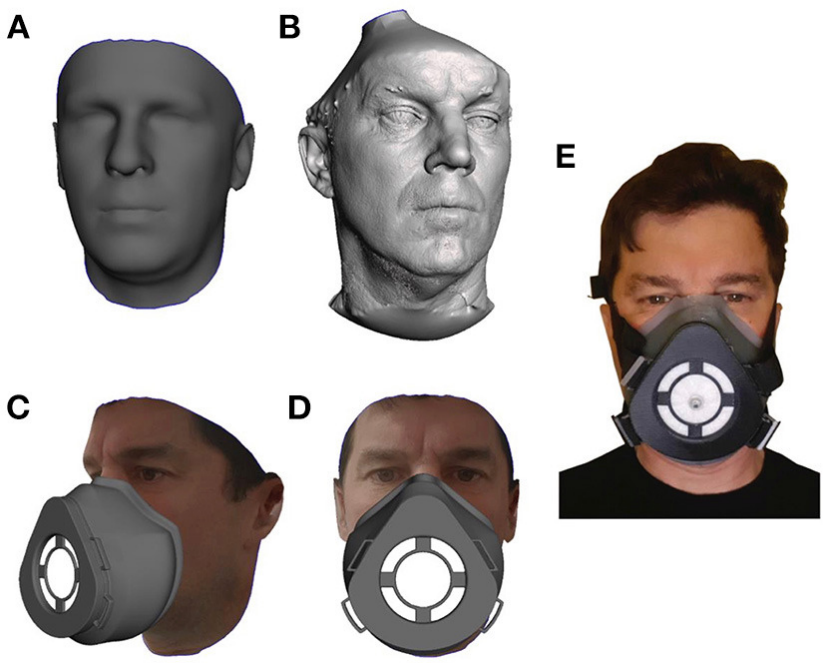
The process of converting an individuals facial scan to a personalized 3D mask is demonstrated. **(A)** Is a 3D facial CAD Model generated using the Crisalix application while **(B)** is a 3D facial CAD Model generated from the Artec Eva scanning process. Both models are shown prior to any manipulation. **(C,D)** The full mask on a participant's virtual face. **(E)** The final 3DPPE mask worn by the participant.

The standard respirator section was designed in Solid Edge (Siemens, Texas, USA) and then combined with the customized section to create the complete respirator. Figure 1A shows a participant's.stl file generated using the Crisalix system and in Figure 1B the same individuals.stl file created using the Artex Eva 3D system. Figures 1C,D shows the simulated respirator mounted on the model, and the filter can be seen in white. Figure 1E shows the complete respirator strapped up and mounted on a participant's head with the filter pierced with a valve prior to a fit test being carried out.

To determine virus viability on the different potential materials for 3D-printing, numeric data were plotted as log^10^-transformed values [assigning a value of 10 min to the “0” h time point] and analyzed by linear regression [for visual display], while the raw data were analyzed by non-linear regression and a one phase decay model to directly estimate half-lives. All analyses were carried out in Graphpad Prism 5™.

Fit test results were analyzed using McNemar's test for paired data. R-COMFI scores were assessed for normality and equivalence of variance using a Shapiro-Wilk test and Levene's test, respectively. Normal datasets of equal variance were analyzed using Student's *T*-test. Normal datasets of unequal variance were analyzed by Welch's unequal variance *T*-Test. Non-parametric data was analyzed using a Mann-Whitney test. MRhyme scores were compared using a one-way ANOVA. All analyses were performed in R Studio (Massachusetts, USA).

## Results

### Quantitative PortaCount face fit testing

**Figure 2:**
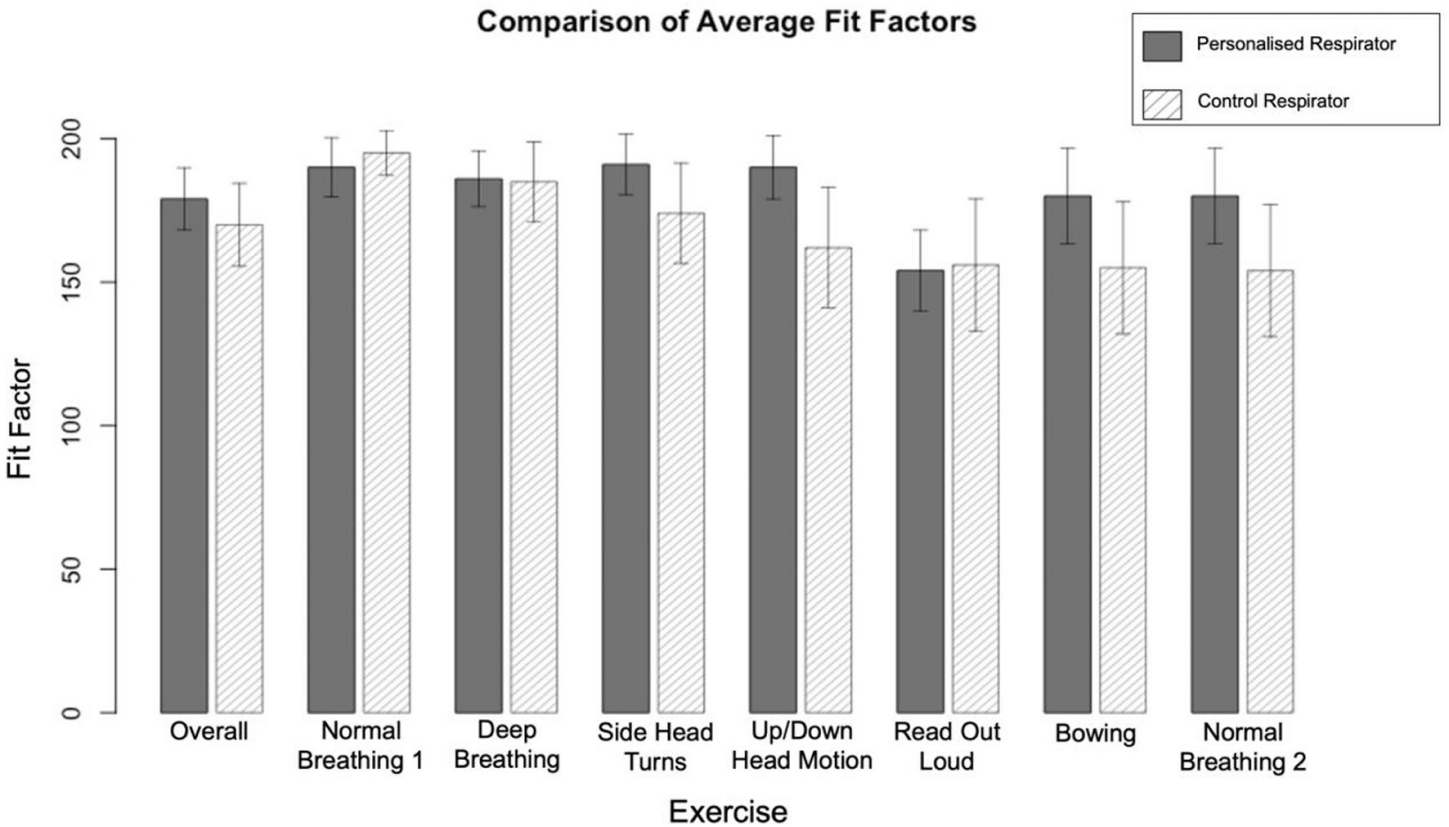
A comparison of the average scores by exercise for the Alpha Solway 3030V and 3DPPE Fit Testing. The maximum possible fit factor was 200, with a minimum of 100 required to pass. Error bars indicate 95% confidence intervals.

In the control group, using the Alpha Solway 3030V FFP3 rated respirator, 38 passed the quantitative fit test, while 12 failed. In the test group, using the personalized 3D printed respirator, 44 passed and six failed (Tables 2, 3 in Supplementary material). As paired data, 11 who had failed the control respirator, passed with the 3D printed respirator. Conversely, five who passed with the control respirator, failed with the 3D printed respirator. Using McNemar's test, there was no significant difference in performance between these groups (*p* = 0.21), confirming that the 3D printed respirator performed as well as the FFP3 control respirator. Both respirators had uniformly high fit factors across all tasks, however the more dynamic tasks had lower scores (Figure 2).

**Figure 3:**
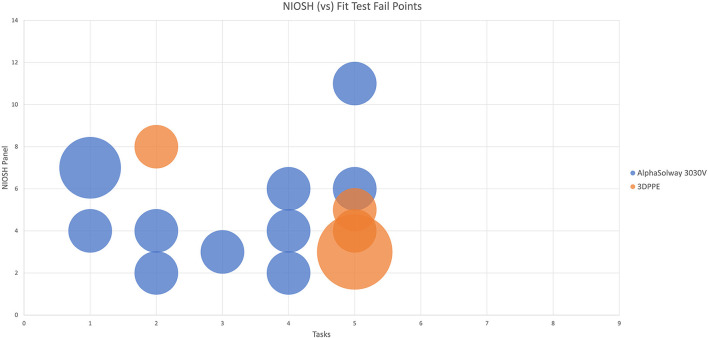
Representation of location of the fit test fail points of each the Alpha Solway 3030V and the 3DPPE masks in comparison to the participants NIOSH Bivariate Panel value.

One way of assigning the a fitting respirator to the correct sized face is through the use of the NIOSH Bivariate Panel (19) where each numbered grid refers to a specific facial width and facial length. Our data in Table 4 in Supplementary material shows the participants' NIOSH panel distribution and correspondingly in Figure 3 the task fail points to participants NIOSH values are mapped. It can be seen that for the Alpha Solway 3030V respirator the failure points were distributed over a wide range of exercises and NIOSH grid points, while with the 3DPPE the fail points were clustered around task five (reading a passage out loud). Notably there was a cluster of failures of those with smaller faces, NIOSH panels 1–4, wearing the control FFP3 respirator failing across several tasks, while those with smaller faces using the 3DPPE failed in the reading out loud task.

### Comfort assessment

Fifty participants completed fit testing for both respirator types. Of these, 47 completed R-COMFI questionnaires after completing the simulated clinical scenario for both respirator types. Following at home wear, 29 responses were received for the personalized respirator, and 34 for the control. As the R-COMFI assesses negative aspects of respirator tolerability, a lower R-COMFI score indicates a more tolerable respirator.

**Figure 4:**
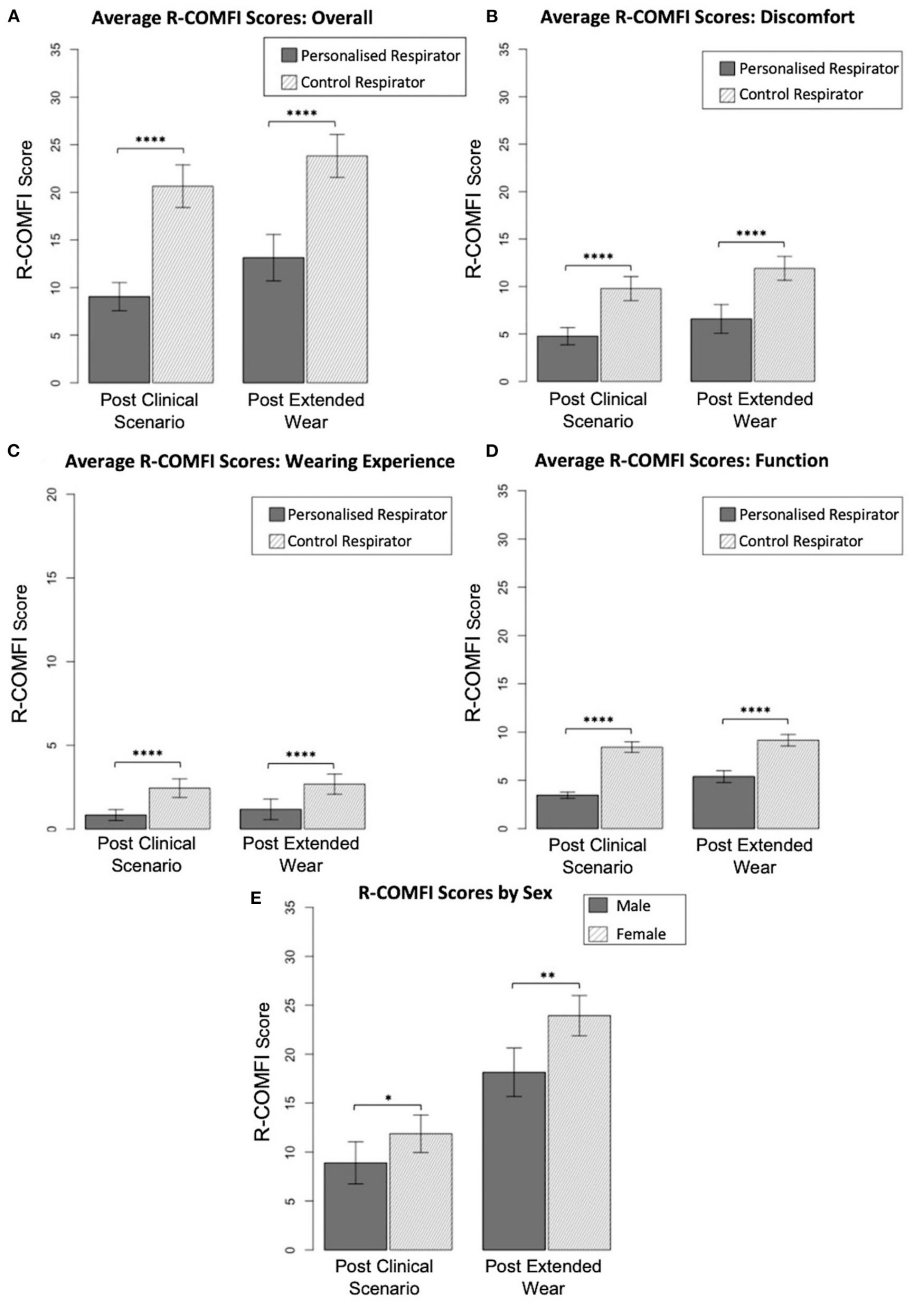
Comparison of R-COMFI scores. **(A)** Overall R-COMFI scores for personalized vs. control respirators following simulated clinical scenario and extended at home wear. **(B)** Comparison of discomfort subsection scores. **(C)** Comparison of wearing experience subs subsection scores. **(D)** Comparison of function subsection scores. **(E)** Sex differences in R-COMFI score for personalized vs. control respirators. Error bars represent 95% confidence interval. *P* < 0.05 are denoted by “*,” <0.01 by “**,” and <0.0001 by “****.”

Mean overall R-COMFI scores following the simulated clinical scenario were significantly lower for the customized respirator than the control respirator (*p* < 0.0001) indicating superior comfort. Following extended wear at home, the mean overall scores were higher for both groups, however the customized respirator maintained a significantly lower R-COMFI score than the control respirator (*p* < 0.0001; Figure 4A).

R-COMFI scores were then stratified into subsections assessing discomfort, wearing experience and function. The customized respirator was more comfortable than the control respirator in each of these subsections, with a significantly lower score (*p* < 0.0001 for all comparisons; Figures 4B–D).

As gender discrepancies in fit testing and PPE specificity have been previously reported (8) results were stratified by sex and differences in overall R-COMFI score assessed by Mann-Whitney test. Female participants had higher average R-COMFI scores for both respirators than male participants. However, the difference in average R-COMFI scores between sexes was significantly reduced using the personalized respirator Figure 4E.

### Communication assessment

Mean MRT scores were 98.03 [95% confidence interval (CI) ±0.83) without a respirator, 97.6 (95% CI ±0.82) for the control respirator, and 97.8 (95% CI ±0.52) for the personalized respirator. All scores are out of 100, where a score of 100 indicates perfect intelligibility. Analysis by one-way ANOVA indicated there was no significant difference between the three groups, demonstrating that speech clarity was not significantly impaired by the use of either respirator.

### Virology testing

Virus viability dropped off with time, following an exponential decay curve, so that when data are plotted in log-log format, a straight line can be fitted to provide a visual guide to decay rate (Figure 2 in Supplementary material). The likely influence of environmental conditions (e.g., temperature, humidity) could be inferred from between experiment variability; the estimated half-lives of virus on polystyrene varied between 1 h in the first experiment (Figure 2A in Supplementary material), 1.2 h (Figure 2B in Supplementary material), and 2.6 h (Figure 2C in Supplementary material). However, none of the various 3D printed plastics or the coatings applied to them showed any major differential effect on virus viability, with the log-transformed data showing similar slopes and half-lives not varying substantially from the baseline polystyrene material.

With regards to decontamination, diluted shower gel was the least effective disinfectant on all surfaces, but even this reduced viable virus load by over 90%. Washing up liquid was the most effective agent, removing virus below the limit of detection in all cases. All other cleaning agents worked effectively with Chlor-Clean™, the NHS recommended decontaminant, removing over 99.9% of virus from all 3D printed plastics tested.

### Scanning techniques: 3D scanning vs. 3D photographic reconstruction

Results for fit testing, comfort and speech intelligibility for the personalized respirators were stratified according to the scanning technique used to generate the stereolithography files for modeling. Despite the Artec Eva scanner producing higher fidelity models, no significant differences were found in fit test pass rate (*p* = 0.8, Chi-square test), R-COMFI scores (*p* = 0.08, Student's *t*-test), or MRT scores (*p* = 0.5, Mann-Whitney test) compared to the Crisalix 3D photographic reconstruction, indicating equivalence in respirator function despite fidelity differences between scanning techniques.

## Discussion

Our study has demonstrated that personalized 3D printed respirators can be produced locally, and can perform equivalently to disposableFFP3 respirators in quantitative fit testing. This adds to the growing body of evidence that novel respirators may be able to fill gaps in overstretched supply lines (20), but only once regulatory approval has been passed. Furthermore, this study provides objective evidence, when compared to disposable FFP3 respirators, that personalized respirators are more comfortable, do not impede clarity of speech, increase the users' wearing experience and thereby their function.

Neither 3D printing nor 3D scanning are new, scarce nor expensive technologies in developed economies. Additive manufacturing has become an established technique in industries ranging from the aerospace sector to biomaterial manufacturing. Readily available computing power through ubiquitous mobile phones and cloud-based data processing, enables 3D rendering of objects as complex as the human face in a matter of minutes. Together these technologies enable the creation physical templates that can be used to adapt and manufacture personalized respirators on-demand.

The pervasiveness of such technology has meant several groups have proposed early prototypes of 3D printed respirators. However, their clinical effectiveness has not been demonstrated. Initial attempts to verify function have focused primarily on the fit-test performance, and non-standardized wearing outcomes in static conditions (20). However, a respirator with perfect filtration yet poor user comfort in dynamic environments will quickly be abandoned.

In pressurized environments, maintaining staff comfort is important to improving their overall performance (21). Using a simulated stressful scenario of managing a cardiac arrest in a clinical simulation suite, the comfort and function of the both the personalized and control respirator could be objectively assessed. Coupled with a longer wear at home assessment, participants uniformly reported that the personalized respirator was more comfortable to wear, increasing their ability to conduct tasks without distraction.

Notably, women and non-Caucasians have been documented to have higher failure rates with FFP3 respirators (9). As disposable respirators may have been originally designed for mechanized industrial environments, dominated by a male workforce, their fit may not be appropriate for the healthcare setting which has a higher proportion of female workers. Previous research supports this, finding only 26% of women use PPE designed for them (22). Additionally, differing ethnicities have varying anthropometric facial features requiring differently shaped respirators for a comfortable and safe fit. Whilst this study did not recruit enough participants from different ethnic backgrounds, the data available between males and females shows that personalized respirators were more comfortable for both sexes than the control, and that personalized respirators reduced sex differences in overall comfort compared to the control respirator.

Previous work has shown that healthcare workers feel more protected by reusable elastomeric respirators, however many reject them due to impaired comfort and communication (23). Data presented here however shows that personalized reusable elastomeric respirators can demonstrate improved comfort and communication over single use filtering facepiece respirators. Notably, the personalized respirator outperformed the control in the function section of the comfort assessment tool, which assesses the wearer's perceived impact of respirator use on the quality of team-based care they can provide. This suggests personalized reusable elastomeric respirators can improve subjective clinical performance while reducing the environmental impact of single-use PPE.

This study is limited as we were only able to compare our 3D printed design against disposable FFP3 respirators, and not commercially available “off-the-shelf” elastomeric respirators due to supply issues at the peak of the pandemic, when this study was conducted. Even though our customized respirator performed equivently to the control, there were still smaller faced participants who failed the fit test whist speaking out loud. This was likely due to manufacturing errors, but with more time and resources these respirators could be adapted to improve fit, especially during speaking. Additionally, as this was the first assessment of the 3D-printed design, further rigourous testing in line with national and international regulators would be necessary to determine if 3D-printed customized respirators are safe enough to deploy to healthcare staff in hazardous environments.

We aimed to use resources easily available to healthcare staff to produce 3D models of their faces, whether that was through existing 3D face scanners in the local medical photography department, or by simply uploading three self-generated photographs from their phones. Our results demonstrate, that both techniques were equivocal in producing respirators that were personalized to individuals and performed equally across all outcome measures. This is impactful, as means that hospitals do not necessarily need to acquire new hardware to facilitate personalized respirator production, especially if working with a local university partner. Additionally, the use of personal phones allows healthcare staff to be in control of data they share, and provide this in a socially distanced manner, avoiding unnecessary trips to the hospital, a key feature of the pandemic.

## Conclusion

Our study has demonstrated that novel 3D printed personalized respirators performed as well as the highest rated disposable FFP3 respirators used by front-line healthcare workers. In addition, this work has confirmed that personalized respirators are more comfortable to wear in stressful situations and during longer wear than available disposable respirators. These benefits were achieved with no sacrifice in terms of communication. Finally, the materials were easily decontaminated of viral particles using readily available detergents and disinfectants used in hospital settings. Altogether, this study provides evidence that personalized 3D printed respirators may be a reusable alternative to existing FFP3 supplies, particularly during times of supply chain pressures, once regulatory approval has been sought and gained.

## Data availability statement

The original contributions presented in the study are included in the article/Supplementary material, further inquiries can be directed to the corresponding author.

## Ethics statement

This study was approved by the local institutional review board, NHS Lothian Research and Development Office (Project No: 2020/0112 and Sponsor Reference: AC20052). The patients/participants provided their written informed consent to participate in this study. Written informed consent was obtained from the individual(s) for the publication of any potentially identifiable images or data included in this article.

## Author contributions

AR, KS, and AS conceived the work and designed the study with AM, JN, and PD. AR, KS, AM, AL, AC, AAC, KD, and ST acquired the data and analyzed with GC, KT, PD, and JN. AS was the principal investigator for the grant and as the guarantor for the work, agreed to be accountable for all aspects of the work, including integrity, and accuracy. AR, KS, and AL were the plastic surgery clinicians responsible for participant recruitment and overall management of the project. AM, AS, KD, AC, and AAC were the engineering team responsible for respirator design and manufacture. KT and GC were the speech and language therapists responsible for analyzing and interpreting speech data. JN was the statistician responsible for statistical design. PD and ST were the virology team, responsible for virology experiments and analysis. All authors interpreted, drafted, and revised the work critically before final approval. All authors contributed to the article and approved the submitted version.

## Funding

This work was supported by the Chief Scientist Office of Scotland as part of the Rapid Research in COVID-19 programme, Grant No. COV/EDI/20/13, and approved by the local institutional review board, NHS Lothian Research and Development Office [Project No: 2020/0112 and Sponsor Reference: AC20052]. All study authors were independent of the funders. Additionally, resources for AS, AM, KD, and AC were supported by CDT Capital Equipment Grants [EP/L016834/1], EPSRC ORCA Hub [EP/R026173/1], and Connect-R: Providing Structure in Unstructured Extreme Environments [Innovate UK (TS/S017623/1)]. AR was supported by the Starter Grant for Clinical Lecturers [Academy of Medical Sciences, SGL021/1004]. PD and ST were also supported by Institute Strategic Programme funding [BB/P013740/1] from the British Biotechnology and Biological Sciences Research Council.

## Conflict of interest

The authors declare that the research was conducted in the absence of any commercial or financial relationships that could be construed as a potential conflict of interest.

## Publisher's note

All claims expressed in this article are solely those of the authors and do not necessarily represent those of their affiliated organizations, or those of the publisher, the editors and the reviewers. Any product that may be evaluated in this article, or claim that may be made by its manufacturer, is not guaranteed or endorsed by the publisher.
