# Supplementary material for: Personalised 3D printed respirators for healthcare workers during the COVID-19 pandemic
Source: Front Med Technol. 2022 Aug 1;4:963541. doi: 10.3389/fmedt.2022.963541 (PMC9380470; doi:10.3389/fmedt.2022.963541)
Supplement: Supplementary file 1 [file Data_Sheet_1.pdf]

Supplementary Material

Supplementary Figures and Tables

Figures

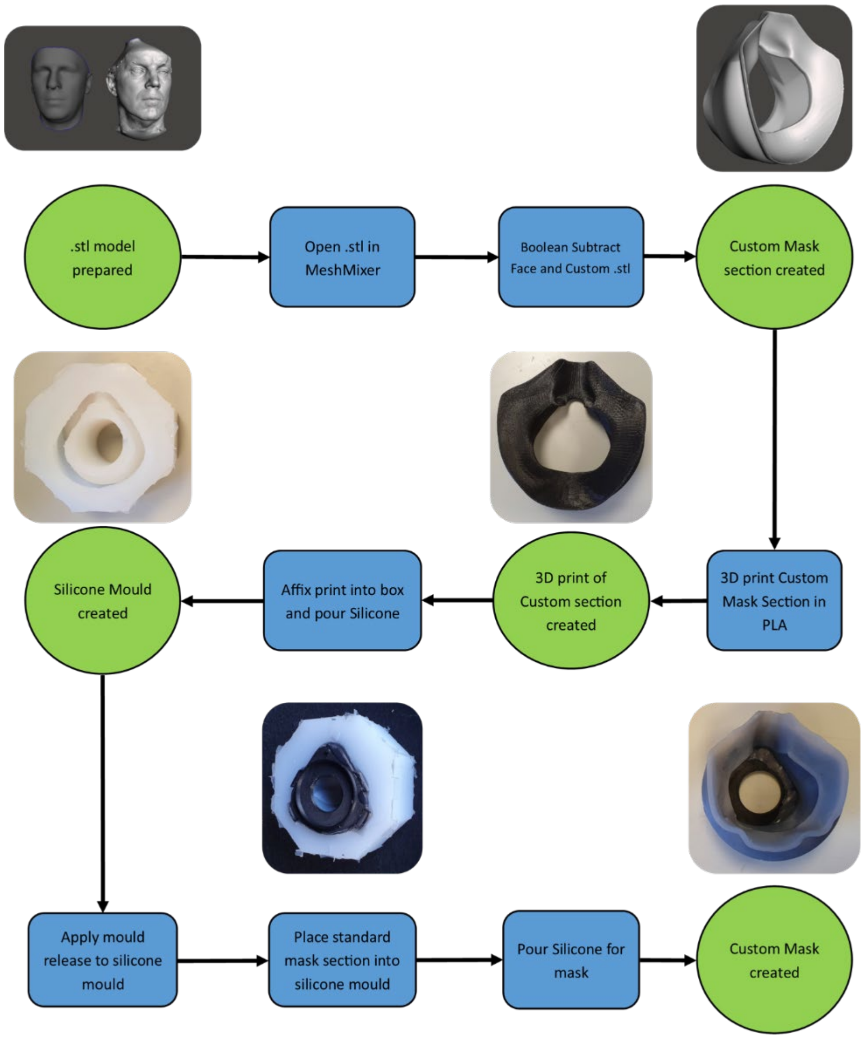

Supplemental Figure 1: Flow diagram of the manufacturing process of the customised 3D-printed respirator.

Tables

Anthropometric Data	Female Number	Male Number	Face Width Mean (±SD)	Face Length Mean (±SD)	Eye Width Mean (±SD)	NIOSH Panel Mean (±SD)
3D facial scanning	10	17	141(±8.5)	119 (±8.1)	62 (±4.1)	5(±2.2)
3D photo reconstruction	11	12	132 (±4.6)	117 (±5.9)	60 (±3)	4(±2)

Table 1. Anthropometric data of the participants who completed the study.

Respirator		Overall Fit Factor	Normal Breathing 1	Deep Breathing	Side Head Turns	Up/Down Head Motion	Read Out Loud	Bowing	Normal Breathing 2
Alpha-Solway	Mean	170.2	195.3	185.7	174.3	162.8	156.8	155.8	154.6
	SD	52.4	28.5	50.2	63.9	76.3	83.3	83.0	83.5
3DPPE	Mean	179.5	194.3	186.2	191.8	190.6	153.1	176	167.8
	SD	39.2	29.2	35.2	38.1	41.0	54.6	65.8	66

Table 2. Overall fit factor, and individual fit-factors during each task of the Portacount quantitive fit test.

	Overall Fit Factor	Normal Breathing 1	Deep Breathing	Side Head Turns	Up/Down Head Motion	Read Out Loud	Bowing	Normal Breathing 2
Alpha-Solway								
	58	201	201	201	18	0	0	0
	173	201	201	201	201	201	201	94
	35	35	0	0	0	0	0	0
	110	201	76	0	0	0	0	0
	96	201	201	47	0	0	0	0
	80	80	0	0	0	0	0	0
	86	201	201	201	0	0	0	0
	86	201	201	201	0	0	0	0
	28	201	0	0	0	0	0	0
	114	201	201	201	201	0	0	0
	57	201	201	0	0	0	0	0
	114	201	201	201	201	0	0	0
3DPPE								
	156	201	186	201	201	85	0	0
	25	201	190	201	201	25	0	0
	25	25	0	0	0	0	0	0
	147	201	126	201	198	91	0	0
	129	201	201	201	201	53	0	0
	68	104	106	40	0	0	0	0

Table 3. Failure points for participants who did not pass the quantitative fit test.

Anthropometric	
Face Width mean (SD), mm	136.99 (8.21)
Face Length mean (SD), mm	118.26 (7.24)
**NIOSH panel**	
1 no./total (%)	3/50 (6%)
2 no./total (%)	3/50 (6%)
3 no./total (%)	10/50 (20%)
4 no./total (%)	8/50 (16%)
5 no./total (%)	3/50 (6%)
6 no./total (%)	8/50 (16%)
7 no./total (%)	9/50 (18%)
8 no./total (%)	3/50 (6%)
9 no./total (%)	2/50 (4%)
10 no./total (%)	0/50 (0%)
NA no./total (%)	1/50 (2%)

Table 4. The breakdown of the participants mean face width and length are shown, followed by a breakdown of the particpants distribution over the NIOSH Bivariate Panel.

Supplementary Data

Virology Detailed Methods

The project was prompted by the Severe Acute Respiratory Syndrome Coronavirus-2 (SARS-CoV-2) but for biosafety, cost, and time constraints we used a vaccine strain of influenza A virus (IAV) as a surrogate. Like SARS-CoV-2, IAV is an enveloped virus with a lipid envelope spread by respiratory and fomite transmission routes (13) and is thus likely to show similar sensitivities to SARS-CoV-2. It is also a medically important pathogen in its own right that has caused multiple pandemics over the last century, which we have prior expertise in measuring surface survival and disinfection strategies (14).

Viral stock: H1N1 strain influenza A virus A/Puerto Rico/8/1934 (PR8) was grown in embryonated eggs as previously described (15), diluted in Dulbecco's modified Eagle's medium (DMEM; Sigma, Dorset, UK) supplemented with 1% bovine serum albumin (BSA) to a titre of around 10^8 plaque forming units (PFU)/ml and snap frozen in aliquots at -80°C. On the day of experiment, virus was thawed on ice.

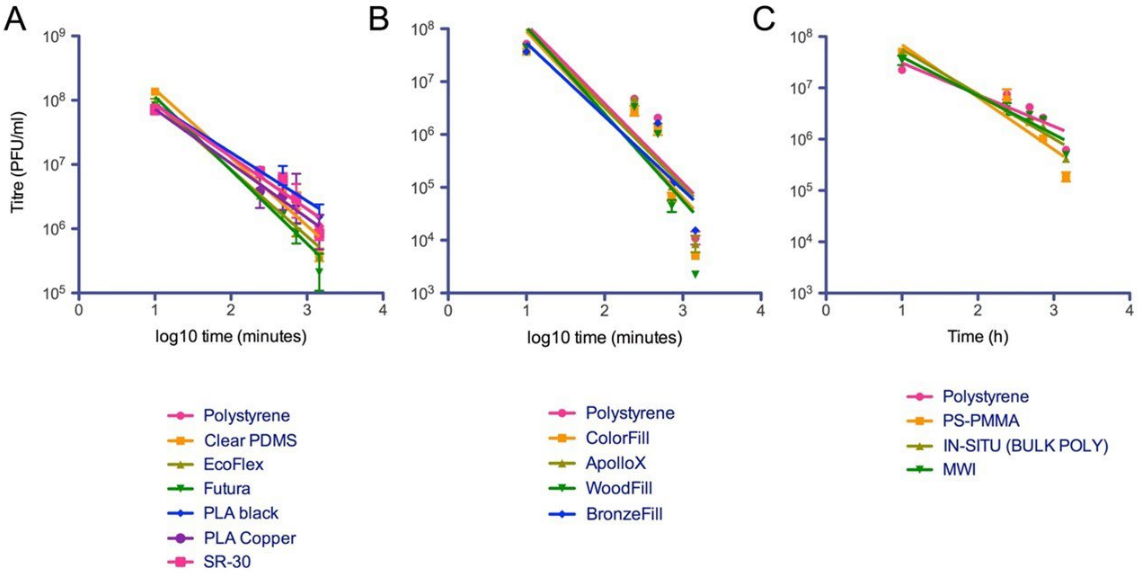

Supplemental Figure2: Effect of plastic type on virus viability. Samples of the indicated plastic materials were inoculated with IAV and virus viability measured by plaque assay at 0, 4, 8, 12 and 24h. Data are the Mean ±SEM of 3-5 replicates plotted as log^{10}- transformed values and analysed by linear regression.

Tissue culture and plaque assays: Madin Darby canine kidney (MDCK) cells were maintained in DMEM supplemented with 10% fetal bovine serum (Gibco), 1x Penicillin/Streptomycin and 1x L-Glutamine (Thermo Fisher Scientific, Massachusetts, USA) at 37°C in 5% C02. For plaque assay, MDCK cells were plated in 6-well plates and allowed to grow to confluency. Before virus inoculation, medium was removed and cells washed with phosphate-buffered saline (PBS). Viral samples were serially 10-fold diluted (down to 10^{-7}) in serum-free DMEM, before 500µl of the 10^{-2} to 10^{-7} dilutions were added to the plates. After a 1-hour incubation at 37°C, cells were overlaid with 2 ml of SFM supplemented with 1.2% Avi-cell RC-581, 0.14% BSA, and 1µg/ml L-(tosylamido-2-phenyl) ethyl chloromethyl ketone treated trypsin and incubated for 48 hours to allow viral plaques to develop.

Cells were then fixed in 10% formaldehyde in PBS and stained with toluidine blue dye to visualise plaques. For each plate, the well with between 10-100 plaques was counted and the corresponding viral titre (in PFU/ml) was calculated.
